# An Ounce of Prevention: Coronavirus (COVID-19) and Mass Gatherings

**DOI:** 10.7759/cureus.7345

**Published:** 2020-03-20

**Authors:** Allan R Escher

**Affiliations:** 1 Anesthesiology/Pain Medicine, H. Lee Moffitt Cancer Center and Research Institute, Tampa, USA

**Keywords:** coronavirus, covid-19, world health organization, mass gathering, thermal scanners, religious pilgrimage, olympics, 2019-ncov, ebola, iso 31000

## Abstract

Widespread, non-stop, and often sensational coverage of the coronavirus (COVID-19) has caught many governments flat-footed in efforts to protect the health and safety of their citizens. In response to the current global health event, the World Health Organization (WHO) declared COVID-19 a pandemic. Mass gatherings present a historic challenge in protecting the health and safety of attendees. The majority of the prominent mass gatherings are religious in nature. Global sporting events, such as the Olympics and the World Cup, pose unique health risks to attendees and host nations. Deferment or cancellation of such mass gatherings may exert an extraordinary economic loss to the host nation. Universal adoption of best practices for infection control is the surest way for governments to prepare for mass gatherings. In these uncertain times, it is up to intergovernmental organizations to be the voice of reason.

## Editorial

The novel Coronavirus (2019-nCoV) came to world attention in December 2019 in Wuhan, China; the World Health Organization (WHO) designated it a pandemic on March 11, 2020 [1]. At first, many individuals and nations assumed that it would be contained within China due to rapid implementation of quarantine for the city of Wuhan and the remarkable construction of two hospitals in only 12 days. The zoonotic transmission of COVID-19 from wildlife to humans led to a Chinese ban on wildlife trade; however, the rapid spread of COVID-19 shows a systematic and expeditious transmission between humans [2].

**Figure 1:**
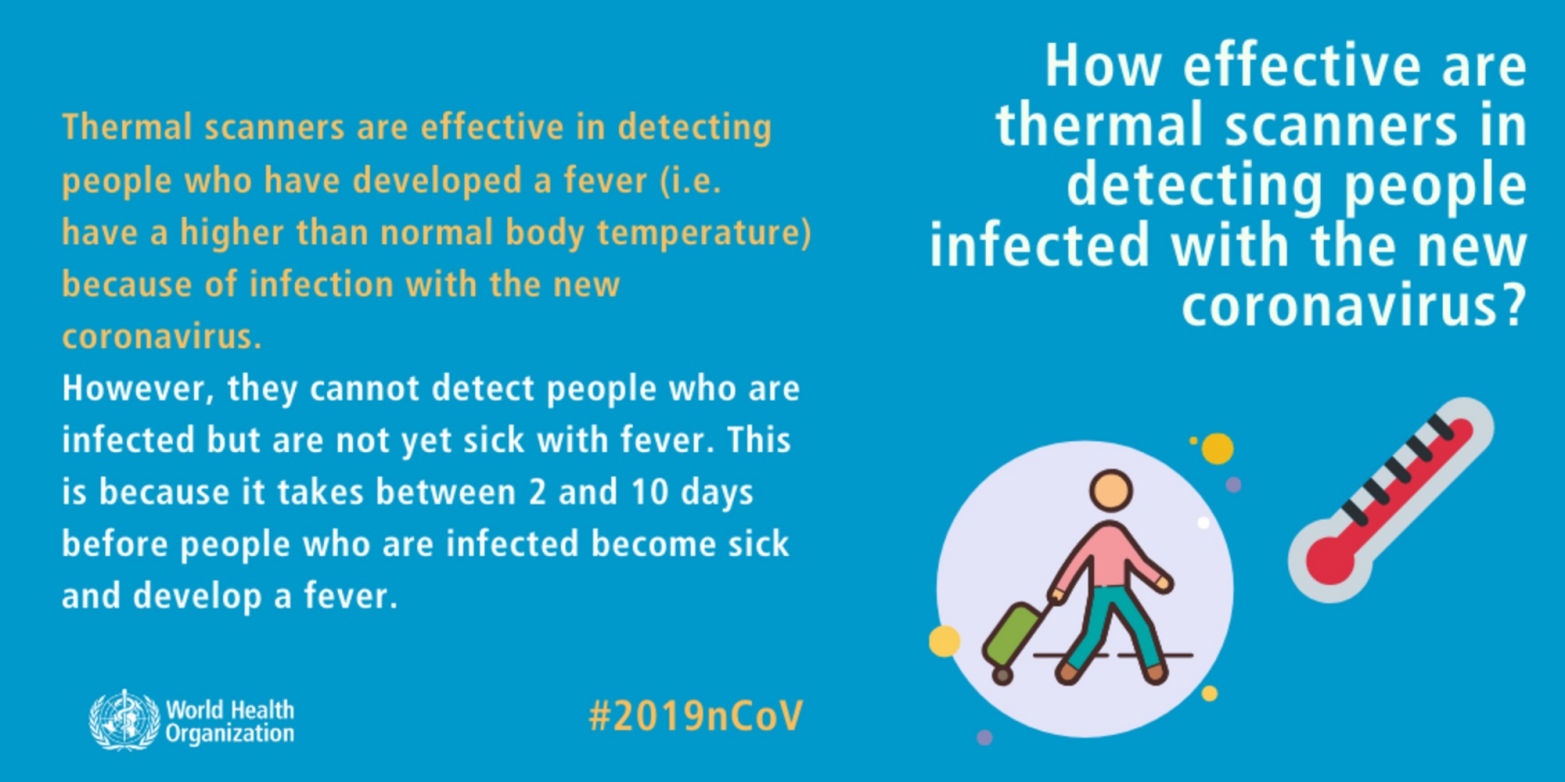
WHO - How effective are thermal scanners in detecting people infected with the new coronavirus? WHO, World Health Organization

As of this writing, the current number of infected is 153,648, along with 5,746 deaths and cases in 146 countries, areas, or territories [1]. Governments have been hesitant in their response with border and school closures, bans on public gatherings, and screening of passengers at transportation hubs. The question remains whether this is truly effective. Some authorities have advocated the usage of thermal scanners to identify those who are febrile. These scanners could be used today to identify febrile individuals who will be in close contact with heads of state, government, or business. Although well-intentioned, this is imprecise for mass gatherings. As seen below, many infected, yet non-febrile individuals will be missed with such screening tools (Figure 1).

In the current climate, it is essential to take a common-sense approach to mass gatherings. The WHO defines a mass gathering as a “concentration of people at a specific location for a specific purpose over a set period of time which has the potential to strain the planning and response resources of the country or community” [3]. Examples of sporting events include the 2020 Olympics in Tokyo or the 2022 World Cup in Qatar. Religious pilgrimages such as the Hajj in Islam and Lourdes in Catholicism draw millions of pilgrims every year. The triennial Kumbh Mela, the Hindu religious pilgrimage festival, can draw up to 120 million people over two months [4]. The health and safety of vulnerable populations in such events are quite challenging.

In response, the WHO recently published, "Key planning recommendations for Mass Gatherings in the context of the current COVID-19 outbreak (Interim guidance)" [1]. This technical guidance is a revision of the prior documents: "Mass gatherings in the context of pandemic (H1N1) 2009 influenza" and "International meetings attended by individuals from Ebola virus disease-affected countries." [1} The WHO Guidelines are a comprehensive resource for governments on how to plan for the myriad logistics of mass gatherings. Risk assessment, response, and surveillance are cornerstones of preparation; the International Organization for Standardization (ISO) 31000 is a well-accepted approach to risk management [3]. Recent consequences of this standard have resulted in the cancellation of mass gatherings in Lourdes and the closure by Saudi Arabia of pilgrims to Umrah. It remains to be seen if the Olympics will proceed as scheduled or deferred until a later date. 

The Ebola experience merits special consideration. After COVID-19 patients have successfully recovered, there needs to be surveillance of patients who are cured. Caution must be exercised in deeming a patient "virus-free." During the Ebola outbreak, some patients were declared “virus-free”; subsequently, the non-transmissible, but present, Ebola virus was detected in the immune‐privileged eye [2]. This is but one example of the difficulties faced by public health officials in treating COVID-19 patients.

Governments are tasked with three critical duties in the response to COVID-2019: the coordination of services with access to COVID-19 testing kits, medical supplies and equipment, accurate and timely communication, and maintaining public trust in their government [5]. Intergovernmental organizations serve as valuable resources in a pandemic. The WHO has a variety of tools to assist governments in their response to COVID-19. These include technical guidance such as “Critical preparedness, readiness and response actions” and “Responding to community spread of COVID-19” [1]. It is up to the host nation, however, to educate its population on best practices for infection control: consistent hand hygiene, social distancing, respiratory hygiene, testing, and the use of quarantine.

With adherence to the existing WHO mass gathering guidelines, governments have a narrow window to mitigate the spread of the novel Coronavirus (2019-nCoV), optimize their healthcare system, and maintain the people's trust in their government.

## References

[1] (2020). Coronavirus disease (COVID-19) outbreak. https://www.who.int/emergencies/diseases/novel-coronavirus-2019.

[2] Ashour HM, Elkhatib WF, Rahman Rahman, MM MM, Elshabrawy HA (2020). Insights into the recent 2019 novel Coronavirus (SARS-CoV-2) in light of past human coronavirus outbreaks. Pathogens.

[3] World Health Organization (2020). Public Health for Mass gatherings: Key Considerations. Travel Med Infect Dis.

[4] Memish ZA, Steffen R, White P, Dar O, Azhar EI, Sharma A, Zumla A (2020). Mass gatherings medicine: public health issues arising from mass gathering religious and sporting events.. Lancet.

[5] Legido-Quigley H, Asgari N, Teo YY (2020). Are high-performing health systems resilient against the COVID-19 epidemic?. Lancet.

